# Effectiveness of dietary interventions among adults of retirement age: a systematic review and meta-analysis of randomized controlled trials

**DOI:** 10.1186/1741-7015-12-60

**Published:** 2014-04-08

**Authors:** Jose Lara, Nicola Hobbs, Paula J Moynihan, Thomas D Meyer, Ashley J Adamson, Linda Errington, Lynn Rochester, Falko F Sniehotta, Martin White, John C Mathers

**Affiliations:** 1 Human Nutrition Research Centre, Newcastle University, Newcastle upon Tyne NE4 5PL, UK; 2 Institute for Ageing and Health, Newcastle University, Newcastle upon Tyne, UK; 3 Institute of Health and Society, Newcastle University, Newcastle upon Tyne, UK; 4 Institute of Neuroscience, Newcastle University, Newcastle upon Tyne, UK; 5 Centre for Oral Health Research, Newcastle University, Newcastle upon Tyne, UK; 6 Fuse, UKCRC Centre for Translational Research in Public Health, Newcastle upon Tyne, UK; 7 Walton Library, Newcastle University, Newcastle upon Tyne, UK; 8 Centre for Brain Ageing and Vitality, Newcastle University, Newcastle upon Tyne, UK

**Keywords:** Mediterranean diet, Fruit and vegetables, Retirement, Aging, Randomized controlled trial, Systematic review, Meta-analysis

## Abstract

**Background:**

Retirement from work involves significant lifestyle changes and may represent an opportunity to promote healthier eating patterns in later life. However, the effectiveness of dietary interventions during this period has not been evaluated.

**Methods:**

We undertook a systematic review of dietary interventions among adults of retirement transition age (54 to 70 years). Twelve electronic databases were searched for randomized controlled trials evaluating the promotion of a healthy dietary pattern, or its constituent food groups, with three or more months of follow-up and reporting intake of specific food groups. Random-effects models were used to determine the pooled effect sizes. Subgroup analysis and meta-regression were used to assess sources of heterogeneity.

**Results:**

Out of 9,048 publications identified, 68 publications reporting 24 studies fulfilled inclusion criteria. Twenty-two studies, characterized by predominantly overweight and obese participants, were included in the meta-analysis. Overall, interventions increased fruit and vegetable (F&V) intake by 87.5 g/day (*P* <0.00001), with similar results in the short-to-medium (that is, 4 to 12 months; 85.6 g/day) and long-term (that is, 13 to 58 months; 87.0 g/day) and for body mass index (BMI) stratification. Interventions produced slightly higher intakes of fruit (mean 54.0 g/day) than of vegetables (mean 44.6 g/day), and significant increases in fish (7 g/day, *P* = 0.03) and decreases in meat intake (9 g/day, *P* <0.00001).

**Conclusions:**

Increases in F&V intakes were positively associated with the number of participant intervention contacts. Dietary interventions delivered during the retirement transition are therefore effective, sustainable in the longer term and likely to be of public health significance.

## Background

Increased life expectancy has resulted in the rapid growth in the proportion of the oldest old (>85 years), particularly in developed nations [1]. These trends are accompanied by a greater burden of disability, frailty and chronic disease, and greater health care costs [2].

Adopting healthy dietary patterns can reduce morbidity and mortality risk. For example, the so-called ‘Mediterranean’ diet (MD), characterized by higher intake of vegetables, fruit, legumes, cereals and fish, lower intake of meats and dairy products and moderate intake of red wine, is a dietary pattern that is associated with lower risks of all-cause mortality, death from cardiovascular disease (CVD) and cancers, age-related diseases including Parkinson’s and Alzheimer’s diseases, obesity and weight gain [3-9]. Multi-center studies show that country of study and sex do not affect these findings [3,4,10], supporting the hypothesis that the MD benefits may be generalizable. The MD components that are effective in driving these health benefits include moderate consumption of alcohol, low consumption of red meat and meat products, and high consumption of vegetables, fruits, nuts, legumes and fish, and use of olive oil as the main source of fat [11].

Life events represent windows of opportunity in which behavior change interventions may be more effective [12]. Retirement from work is one such life event and the importance of nutritional education during this period has been previously highlighted [13]. The need for interventions in the retirement transition is illustrated by the observed weight gain and greater abdominal obesity in people who retire from active jobs and decrease their consumption of fruit and dietary fiber [14]. Such increases in body weight in mid-life predict poorer health in later life [15]. The limited evidence available indicates that retirement may have divergent effects on food intake and that economic factors may be important determinants of dietary choices at this life-stage [16]. In addition, the single study that has evaluated behavioral interventions aimed at improving physical activity and adopting a low energy density diet in recently retired individuals [17] found that these behaviors improved slightly, but not significantly, in the intervention group. Given the lack of interventions specifically targeting retirement, a critical analysis of the evidence on the effectiveness of dietary interventions focusing on those of retirement age should offer evidence of likely benefits to health at this key life-stage.

This systematic review and meta-analysis aimed to assess the effectiveness of dietary interventions that promote a health dietary pattern such as the MD, or any of its component food groups, among adults in the retirement transition age range, with dietary behavior change as the primary outcome. We also aimed to identify characteristics of effective interventions.

## Methods

Our systematic review was conducted according to Cochrane [18] and the Centre for Reviews and Dissemination guidelines [19] and is reported according to PRISMA guidelines (Additional file 1: Figure S10) [20]. The protocol has been registered with PROSPERO, the International Prospective Register of Systematic Reviews (Registration number CRD42011001484).

In April 2013, 12 electronic databases were searched systematically from inception: Medline, Embase, PsycInfo, Scopus, Web of Science, CINAHL, ASSIA, Cochrane Database of Systematic Reviews, CAB Abstracts, Conference Papers Index, WorldCat Dissertations database and Index to Theses. Reference lists of identified publications and previously published related systematic reviews were hand searched to identify other studies potentially eligible for inclusion.

The search strategy involved combining words from the following three concepts: 1) diet; 2) randomized controlled trials (RCTs); 3) people in the retirement transition or of a relevant age. Prior to searching, reviewers carried out an extensive exercise to identify relevant terms. The search terms were translated into a search strategy using a combination of index terms and keywords, which was refined iteratively in response to emerging results. Highly sensitive search filters for identifying RCTs were used in Medline and Embase [18]. The final list of search terms for Medline is provided in Additional file 1: Box S1. This search strategy was adapted as necessary for additional databases and is available on request.

### Study selection criteria

Only RCTs, including cluster-RCTs, were included. To identify interventions carried out in populations with standards of living similar to the UK, only publications from most developed countries in the United Nations Index [21], with an English language abstract were included.

Given the heterogeneity in age at retirement, here we defined the retirement transition period as age 54 to 70 years and sought RCTs of adult participants with sample mean or median ages between 54 and 70 years. Studies involving non-institutionalized adults with or without health risk factors (such as overweight, abdominal obesity, raised blood pressure, abnormal lipid levels and metabolic syndrome) were included.

### Characteristics of interventions

We searched for interventions promoting healthy dietary patterns such as the MD, as well as interventions promoting any of its component food groups (that is, increased fruit and vegetables (F&V); legumes or pulses; nuts and seeds; unrefined cereals; olive oil; fish; moderate consumption of wine; low consumption of meat and meat products). Studies investigating lifestyle interventions including other components, such as physical activity, were included only if the effects of the diet component were reported independently. To assess sustained impacts on behavior change, only interventions with a follow-up of more than three months were included.

Studies promoting other dietary patterns (for example, vegetarian), laboratory feeding trials (not intended to assess behavior change), studies on change in a specific macro/micro nutrient (for example, low-fat, high-protein), studies promoting pre-fabricated diet foods or meal-replacement drinks, and studies testing dietary supplements (for example, fish oil) were excluded.

### Outcome measures

Our primary outcome was dietary change defined as: change in consumption of one or more components of the MD; improved MD adherence at follow-up as assessed by established MD-scores [22]; and quantitative analysis of MD components (for example, change in the number of portions, servings or weight consumed of fruit and/or vegetables, fish, olive oil or red meat). The method of outcome measurement was documented (for example, diet recalls, food diaries and food frequency questionnaires (FFQ)). Outcomes reported as number of portions/servings were converted into grams using the portion size reported in the original articles. However, for studies originating from the USA and not declaring portion sizes, we converted these data using the US Department of Agriculture standard portion sizes [23] assuming the following portion sizes: F&V 113 g, fish 110 g.

### Data extraction

Two reviewers independently assessed publications for eligibility. The decision to include studies was hierarchical and made initially on the basis of the study title and abstract; when a study could not be excluded with certainty at this stage, the full-text was obtained for evaluation. A standardized, pre-piloted form was used to extract data from the included studies for assessment of study quality and evidence synthesis. Discrepancies between reviewers were resolved through discussion with a third reviewer and a consensus approach was used. Extracted information included: study design (country, methods of recruitment, follow-up length, methods of analysis, completion rates, number of intervention contacts – that is, contacts during delivering the intervention rather than contacts when only measurements were taken); participants characteristics (population and setting, inclusion/exclusion criteria, baseline characteristics); description of measurement methods; outcome measures (dietary intake); and information to assess the risk of bias. Study quality was assessed using the Cochrane risk of bias tool [18]. Two reviewers extracted data, one independently and the second confirming or completing information required.

### Statistical analysis

Review Manager (RevMan Version 5.1 for Windows Copenhagen: The Nordic Cochrane Centre, The Cochrane Collaboration, 2011) and Stata (Stata/SE 11.2 for Windows; StataCorp LP, College Station, TX, USA) were used to pool and analyze results from the individual studies. Pooled results are reported as mean differences with 95% CIs and with two-sided *P*-values. A random effects model accounting for inter-study variation was used, thereby minimizing potential bias due to methodological differences between studies. Multiple dietary intervention arms from three studies were included in the meta-analysis. As suggested by Higgins *et al*. [18], excessive weightings from “double counts” originating from the “shared” group (that is, the control group) were controlled by splitting the sample size of the shared group into approximately equal smaller groups for the comparisons; the means and standard deviations were left unchanged. When available, we used results from multivariate models with the most complete adjustment for potential confounders reported in original studies.

Statistical heterogeneity was evaluated using the *I*^2^ statistic [18,19]; the 95% CI for *I*^*2*^ were calculated using Higgins *et al*.’s method [24,25]. Where *I*^2^ was >50%, the degree of heterogeneity was considered high. We performed subgroup analysis to investigate the effects of body mass index (BMI) categories (that is, obese: BMI ≥30; overweight: BMI 25 to 29.9; normal weight: BMI <25 kg.m^−2^. Additional subgroup analyses investigated variables including study size, length of follow-up, participants’ sex and health status, and mode of delivery of intervention, on change in food consumption. In addition, we performed meta-regression analyses to assess the effect of sample size, retention rate, baseline intakes, number of contacts with participants within intervention, and length of follow-up, as continuous variables, on estimates of dietary change.

Publication bias was appraised by visual inspection of a funnel plot of effect size against the standard error (SE), with asymmetry assessed formally with Egger’s regression test [26].

## Results

**Figure 1:**
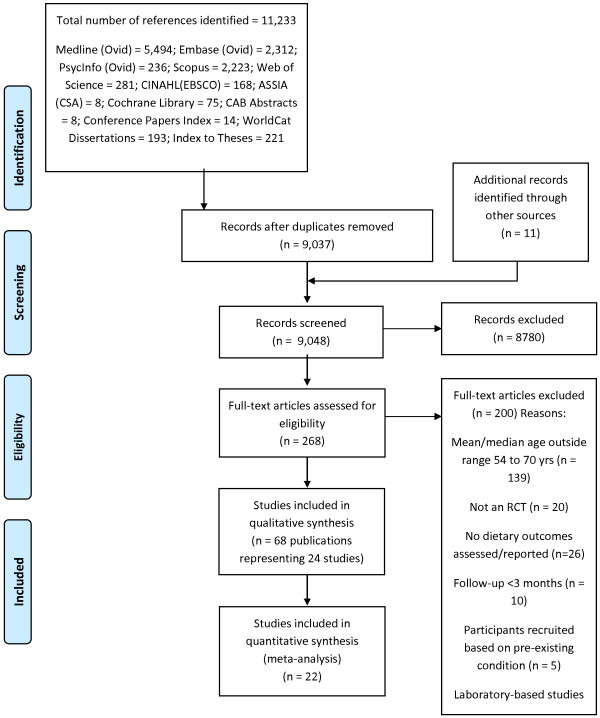
Study selection flow diagram (PRISMA template).

The searches yielded 9,048 publications and results of the screening process are described in Figure 1. Sixty-eight publications reporting 24 studies that met our inclusion criteria were included in the present review; 22 studies provided data for meta-analysis while two studies did not report dietary intake data (Additional file 1: Box S1 and Table S1).

### Study characteristics

Five studies used a cluster RCT design while the remainder used individually randomized, controlled trial designs. The pooled study populations included 63,189 participants who were followed-up for 19 months on average (range 4 to 58 months). The mean ages of the samples in these studies ranged from 54 to 67 years. Four studies recruited women only and two studies men only. Out of 24 studies included in the overall meta-analysis, 14 studies involving 53,987 participants reported a mean BMI ≥25 kg.m^−2^ at baseline thus including a significant proportion of overweight and obese participants, while mean BMI was 23.6 in one study, and 7 studies did not report BMI (Additional file 1: Tables S1-S2).

### Methods of dietary assessment

Most studies used food frequency questionnaires (FFQs) or F&V screener questionnaires to assess self-reported food intake; one study employed a dietary history method [27].

### Dietary intervention

Four studies (from the Netherlands, Spain and Italy) promoting the MD [8,28-30] were identified but one of these did not provide dietary data that could be included in the meta-analysis [30]. One intervention promoted a plant-based diet, 7 interventions promoted a dietary pattern intended to decrease fat intake, and 11 promoted the consumption of F&Vs. These studies employed either a low-fat, or the participants’ usual, diet as the comparator/control group and with follow-up lasting 4 to 60 months. Nineteen studies reported on interventions promoting consumption of F&V or ‘healthier eating’ compared with control groups commonly receiving minimal, or no, intervention. Out of the 22 studies included in meta-analysis, 4 studies had more than one intervention arm. Two-thirds of these studies were carried out in the USA with the others from Japan, Canada, Australia and the UK (Additional file 1: Tables S1-S2).

### Study quality and publication bias

None of the studies satisfied all of the criteria of the quality assessment tool. However, included studies provided an adequate description of methods and randomization procedures, and the average retention rate for the 22 RCTs was 90 ± 10% for all studies, with 5 studies reporting retention rates <80% [29,31-34]. No studies were excluded from analysis based on quality assessment.

**Figure 2:**
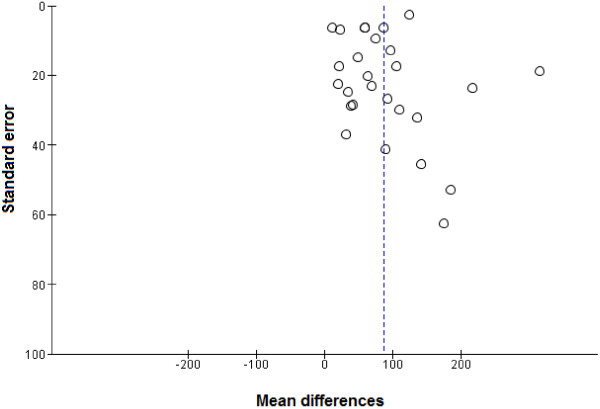
**Funnel plot of randomized controlled trials assessing fruit and vegetable intakes.** Egger’s regression test *P* = 0.394.

A funnel plot of the mean differences in F&V against SEs of all studies did not indicate significant asymmetry, suggesting the absence of publication bias which was supported by Egger’s regression test (*P* = 0.394) (Figure 2).

### Analysis of summary effects of interventions

Two studies assessed the effect of MD interventions using MD scores [8,29]. However, these studies could not be meta-analyzed because different scoring systems (that is, a 14-unit Mediterranean diet score [8] and a Mediterranean adequacy index [29]), were used to report changes in consumption of nuts, vegetables, legumes and fruit. A third intervention reported shifting intakes away from animal sources of fat and protein towards predominantly vegetable sources [30].

**Figure 3:**
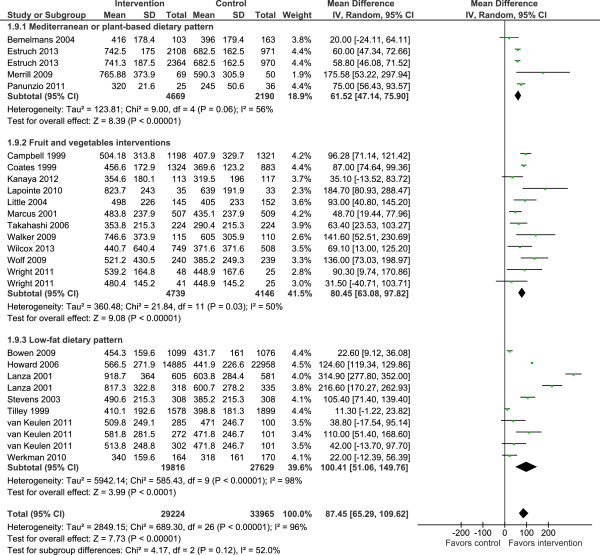
Randomized controlled trials reporting overall fruit and vegetable intakes among people of retirement age.

Consumption of F&V was a common outcome in most interventions and this is the focus of the results described below. The meta-analysis showed that interventions increased F&V consumption significantly (mean difference 87.5 g per day, 95% CI 65.3 to 109.6; *P* <0.00001) with no significant differences when stratifying studies by type of intervention (Figure 3) or BMI (Additional file 1: Figure S1). Overall, heterogeneity levels were high, particularly among the low-fat dietary interventions (Figure 3).

**Figure 4:**
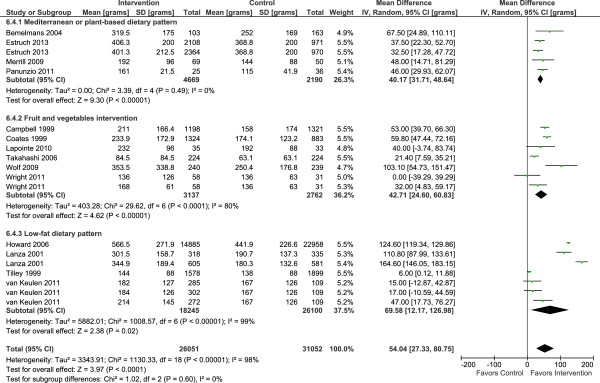
Randomized controlled trials reporting fruit intake among people of retirement age.

**Figure 5:**
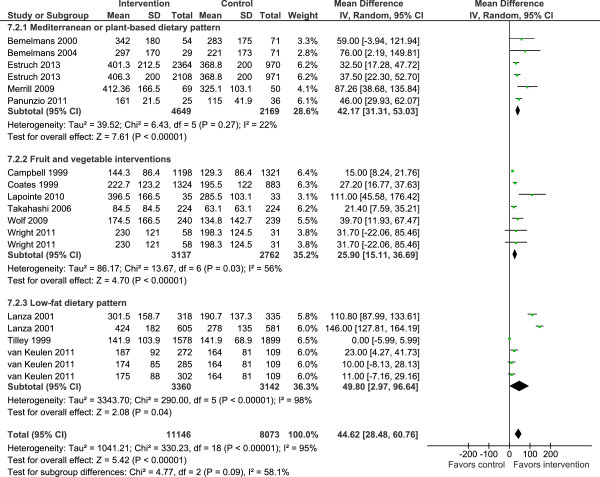
Randomized controlled trials reporting vegetable intake among people of retirement age.

Fourteen of the interventions which reported consumption of fruits and vegetables separately showed significant mean increases of 54.0 g per day (95% CI 27.3 to 80.8, *P* <0.00001) and 44.6 g per day (95% CI 28.5 to 60.8, *P* <0.00001) for fruits and vegetables, respectively (Figures 4 and 5). Adiposity was not associated with changes in intakes of fruits or vegetables in response to intervention (Additional file 1: Figures S2-S3). With the exception of interventions based on the Mediterranean diet, heterogeneity levels were high. Changes in intakes of other food groups were as follows: fish intake was increased significantly by intervention in three studies (mean difference 7 g/day 95% CI 0.5, 12.7; *I*^*2*^ = 93%; 95% CI 85 to 97%) (Additional file 1: Figure S4) [8,28,35,36] while meat intake was decreased significantly in five studies (mean difference -8.7 g/day 95% CI -10.9 to -6.5; *I*^*2*^ = 92%; 95% CI 86 to 95%) (Additional file 1: Figure S5).

### Sensitivity analyses

Sensitivity analysis excluding the largest study [36] did not affect the overall results (F&V mean effect 85.2 g/day, 95% CI 64.1 to 106.3).

### Subgroup analysis

Subgroup analyses (Table 1) showed that in comparison with RCTs in which participants were the unit of randomization, cluster RCTs reported significantly smaller changes in F&V consumption. Further, studies originating from Europe and Asia reported smaller increases in F&V intake than were observed in studies from North America. Subgroup analysis according to sex showed that 16 studies including men and women produced a change in F&V intake of 64.3 g/day with acceptable heterogeneity levels. Single sex studies reported greater changes in F&V consumption, but only four studies or subgroups of men were available and results from these were not statistically significant. Studies using an indirect method of intervention delivery reported smaller changes in F&V intakes in comparison with studies involving face-to-face intervention delivery. Interventions were equally effective in both short-to-medium (that is, 4 to 12 months; 85.6 g/d 95% CI 59.0 to 112.1) and long-term (that is, 13 to 58 months; 87.0 g/day 95% CI 53.2 to 120.9). Subgroup analysis according to the presence of health risk factors (for example, high-cholesterol, high-blood pressure, metabolic syndrome or polyps) vs healthy participants, and by mode of intervention delivery, revealed no significant moderating effects on F&V intakes (Table 1).

**Table 1:** Subgroup analysis of randomized controlled trials reporting overall fruit and vegetable intakes

**Variable (Number of studies or subgroups) [Reference numbers]**	**Mean difference in F&V (95% CI)**	***P *** **(Z-test)**	**Heterogeneity *I***^***2***^**% (95% CI)**
**Study design**			
RCTs (n = 22) [8,27-29,31,33,34,36-45]	97.0 (75.5, 118.6)	<0.0001	94 (93 to 96)
Cluster-RCTs (n = 5) [17,32,46-48]	30.8 (9.9, 51.8)	0.004	67 (26 to 88)
**Ethnicity**			
Mainly Black (n = 4) [32,34,37,43]	82.5 (47.6, 117.4)	<0.0001	61 (0 to 87)
Mainly white Caucasians (n = 23) [8,17,27-29,31,33,36,38-42,44-48]	87.5 (63.8, 112.8)	<0.0001	97 (96 to 98)
**Origin of studies**			
America (n = 14) [31,32,34,36-39,41-43,45-48]	112.6 (77.9, 147.3)	<0.0001	98 (97 to 98)
Europe (n = 9) [8,17,28,29,33,40]	58.1 (45.0, 71.2)	<0.0001	49 (0 to 76)
Asia (n = 3) [27,44]	61.4 (29.4, 93.4)	0.0002	0 (0 to 90)
**Sex**			
Women only (n = 7) [36,38,39,42,45,46,48]	117.6 (75.7 to 159.5)	<0.0001	97 (96 to 98)
Men and women (n = 16) [8,27-29,31-34,37,40,41,44]	64.3 (53.6 to 75.0)	0.0001	38 (0 to 66)
Men only (n = 4) [17,38,43,47]	120.4 (-19.9 to 260.8)	0.09	99 (98 to 99)
**Follow-up time**			
4 to 12 months (n = 23) [17,27,29,31,33,35,37-44,46-49]	85.6 (59.0 to 112.1)	<0.0001	92 (90 to 94)
13 to 58 months (n = 15) [8,17,28,32-34,36-38,45,47]	87.0 (53.2 to 120.9)	0.0001	98 (97 to 98)
**Health status**			
With health risk factors (n = 15) [8,28,31-33,37,38,40,44,47]	82.7 (49.4 to 116.1)	0.0001	95 (93 to 97)
Healthy participants (n = 11) [17,27,29,34,36,39,41-43,45], [46,48]	91.5 (62.7 to 120.4)	<0.0001	96 (94 to 97)
**Mode of intervention delivery**			
Face to face (n = 16) [8,17,27-29,32,36,38-41,43-45,47]	97.6 (69.2 to 125.9)	<0.0001	97 (98 to 99)
Indirect (for example, telephone, post) (n = 10) [31,33,34,37,42,44,46,48]	68.4 (40.8 to 94.5)	<0.0001	64 (29 to 82)

### Meta-regression analysis

**Figure 6:**
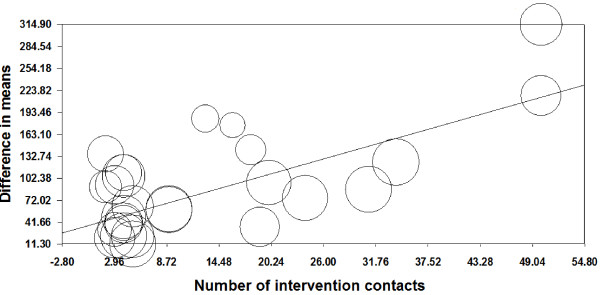
**Meta-regression analysis of effects of number of contacts during interventions on fruit and vegetable intakes.** Slope = 3.80, Q = 36.93, d.f. = 1, *P* <0.0001. The circle size reflects the weight that a study obtained in the meta-regression.

Univariate meta-regression analysis showed a significant positive association between F&V and the number of contacts with participants during the intervention (*P* <0.0001) (Figure 6). However, there were no significant associations between length of follow-up, study sample size or retention rate included as continuous variables, and estimates of change in F&V intake (Additional file 1: Figures S6-S9). Exclusion of studies with higher attrition rates [29,31-34] did not significantly modify the results for change in F&V intake (95.7, 95% CI 65.3 to 126.0; *P* <0.0001; *I*^*2*^ = 97%, 95% CI 97 to 98%). Responses to interventions were unrelated to level of F&V intake at baseline (Additional file 1: Figure S9).

## Discussion

To our knowledge, this is the first systematic assessment through meta-analysis of the effectiveness of dietary interventions attempting to change eating patterns among people of retirement transition age (54 to 70 years). Our meta-analysis of 22 published RCTs involving 63,189 participants demonstrated a moderate but consistently significant (mean 87.5 g/day) increase in F&V consumption in both the short-to-medium term (4 to 12 months) and the longer term (>12 months). The increase in F&V intake observed in these RCTs was positively associated with the number of contacts with participants during the interventions. The increase in F&V consumption was similar in studies involving predominantly overweight or obese participants and in those with and without additional health risk factors. Interventions were slightly more effective in increasing fruit intake (mean 54.0 g/day) than in increasing vegetable intake (mean 44.6 g/day). We found that interventions based on the MD *per se,* as well as interventions promoting other MD-relevant food groups, among people of retirement age, are scarce. Such MD interventions reported results in different ways and meta-analysis of the effect according to Mediterranean diet score [22] was not possible. Although the benefits of the MD in secondary prevention [50-52] are well recognized, only four interventions specifically targeting people at the critical time of retirement transition age were identified. The PREDIMED study [8,53], the largest RCT testing the MD in primary prevention, has shown the effectiveness of the MD in reducing CVD risk and type-2 diabetes and, more recently, peripheral artery disease [8,54,55].

In a previous analysis of studies recruiting mainly younger adults, Brunner *et al.*[56] reported that, compared with no advice, offering any type of dietary advice increased self-reported F&V intake by 1.25 servings/day (95% CI 0.7 to 1.81). As in our study, Brunner *et al.*[56] found greater increases in intakes of fruits than of vegetables. Out of 10 studies examining effects on F&V consumption, Pignone *et al.*[57] found that 30% of studies reported small to no increases (<0.3 servings/day), 50% observed medium increases (from 0.3 to 0.8 serving/day) and 20% reported large increases (1.4 and 3.2 servings/day). Using Pignone’s classification [57], the present meta-analysis showed that 36% of studies reported large increases (that is, >0.8 servings/day), 44% reported medium increases and 20% reported small increases. For the joint WHO/FAO initiative on promoting F&V for health, Pomerleau *et al*. [58] reviewed interventions regardless of study design and reported F&V increases ranging from 0.1 to 1.4 servings/day.

Interestingly, in our analyses, interventions showed no significantly different effect on dietary change in those studies investigating healthy participants than in studies where participants had health risk factors. This suggests that the presence of these risk factors (overweight, abdominal obesity, raised blood pressure or abnormal lipid levels) did not enhance or reduce participants’ responses to the dietary interventions. Interventions delivered indirectly (for example, by telephone) were only slightly less effective than those delivered face-to-face; this finding is supported by recent reports on the effectiveness of telephone-based interventions on physical activity and diet [59,60].

We observed a modest but significant increase in fish consumption, equivalent to an increase of 50 g or 0.35 portions of fish per week [8,28,35,36], which is important because of the beneficial role of fish in reducing cardiovascular risk [61]. In addition, there was a modest, but also significant, reduction in meat consumption, equivalent to a decrease of 60 g of meat per week. However, these findings should be interpreted with due caution because they are derived from relatively few studies with relatively small numbers of participants. This review has revealed the lack of studies which have assessed the effectiveness of interventions promoting other food groups or elements of the MD in the 54- to 70-year age group.

Among the limitations of the present analysis are the high levels of heterogeneity among the studies and hence our findings should be treated with caution. We did subgroup analysis to explore a number of potential sources of heterogeneity; type of intervention, study design, ethnicity, sex, geographic origin of studies and mode of delivery of interventions were identified as sources of heterogeneity. It is of note that the intervention studies included in this review were consistently, and significantly, successful in enhancing F&V intake. Subgroup analysis of 16 studies involving both men and women (total 13,926 participants) showed a mean increase in F&V intake of 64.3 g/day with acceptable heterogeneity levels. In addition, interventions promoting the Mediterranean diet and those interventions which promoted F&V intake *per se* reported similar effect sizes (Figure 3). Single-sex studies were characterized by larger changes in F&V intakes but also by high heterogeneity levels. This subgroup analysis is exploratory and the results obtained should be interpreted with caution because of the small numbers of studies in each sub-group and the *post hoc* nature of the analysis. All studies used self-reported methods of dietary intake (that is, FFQs). The well-recognized limitations of all widely used dietary reporting tools may be amplified when attempting to assess responses to interventions. This is because, by necessity, study participants are aware of the expected dietary behavior and because repeated measurements are burdensome [62], which may introduce reporting bias. Current advances in the development of objective biomarkers of dietary exposure [63,64] may overcome some of these limitations.

The decision to operationalize the retirement transition as a mean or median age in the range 54 to 70 years was based on the wide variability in age at retirement [65]. In the USA, legal retirement age has increased from 65 to 67 since 1960. Age at retirement has also changed in other countries [66]. In the UK, between 2004 and 2010, average age of retirement rose from 64 to 65 for men and 61 to 62 for women [67], whereas retirement age for some employment groups (for example, those in the fire and police services) may be considerably younger, and in other European countries may start as low as the early 50s [68]. Although our review did not assess interventions that focused on retirement *per se* (we found no such studies), we investigated the effectiveness of interventions targeting groups of retirement transition age that is, 54 to 70 years.

The findings of this meta-analysis are striking and show a consistently positive effect of interventions on dietary behavior with respect to F&V consumption across the diversity of RCTs. In addition, the overall good quality of the studies, including low attrition rates, provide confidence in the robustness of the findings.

The results of this review have important public health implications. Our findings are in line with a recent report from the U.S. Preventive Services Task Force (USPSTF) recommendation statements on behavioral counseling to promote a healthful diet and physical activity in adults without pre-existing cardiovascular disease (CVD) or its risk factors [69,70]. The USPSTF reported that behavioral counseling in primary care settings which promoted a healthful diet increased consumption of fruits and vegetables by up to 2.0 servings per day. These results were observed among interventions of moderate to high-intensity, but not low-intensity.

Lock *et al*. [71] estimated that worldwide mortality attributable to low consumption of F&V is 2.6 million deaths/year and raising F&V consumption to 600 g/day could reduce the total worldwide burden of disease by 1.8%. Achieving this dietary target would translate into 31% and 19% lower burden of ischemic heart disease and ischemic stroke, respectively; while for stomach, esophageal, lung and colorectal cancer, the potential reductions would be 19%, 20%, 12% and 2%, respectively. However, there is evidence that changes in F&V intake of the same magnitude as those observed in this review are of public health importance. Results from the European Prospective Investigation of Cancer (EPIC)-Norfolk cohort showed that an increase of 20 μmol/L in plasma ascorbic acid concentration, equivalent to about 50 g per day increase in fruit and vegetable intake, was associated with about 20% reduction in risk of all-cause mortality (*P* <0.0001), independent of age, systolic blood pressure, blood cholesterol, cigarette smoking habits, diabetes and supplement use [72]. More recent results from the EPIC study show that one portion increment in F&V intake was associated with a 4% lower risk of fatal ischemic heart disease (RR = 0.96, 95% CI: 0.92 to 1.00, *P* for trend = 0.03) [73]. In addition, in an RCT with six-month follow-up, an increase in self-reported F&V intake by a mean 1.4 portions (SD 1.7) in the intervention group than in controls, was associated with significant reductions in systolic (difference = 4.0 mm Hg, 95% CI 2.0 to 6.0; *P* <0.0001), and diastolic blood pressure (1.5 mm Hg, 95% CI 0.2 to 2.7; *P* = 0.02) [74]. Recent systematic reviews have estimated the reduction in risk of several common non-communicable, and age-related, diseases associated with increased F&V consumption (Additional file 1: Table S3). These benefits of specific food groups strengthen the evidence for the health-protecting effects of the MD and other healthy dietary patterns [3,4,51,52,75-78]. Such effects are likely to have a significant impact on the burden of chronic diseases, including associated disability and health care costs, among ageing populations. Unfortunately, none of the interventions reviewed included cost or cost-effectiveness analysis of interventions, but such interventions are likely to be highly cost-effective [79].

## Conclusions

In conclusion, this systematic review of RCTs has demonstrated the effectiveness of dietary interventions in increasing F&V and fish intake among adults in the retirement transition age range. Despite the heterogeneity of intervention modalities, F&V intake was increased by approximately 87 g/day and was sustained in the longer term (>12 months). The increase in F&V intake in these RCTs was associated positively with the number of contacts with participants during the intervention indicating that more intensive interventions may offer advantages, although differential cost-effectiveness has not been assessed. These results provide evidence to support the development of interventions to improve dietary behavior at this life-stage to promote health and well-being and to reduce the risk of age-related disease and associated costs to society.

## Abbreviations

BMI: Body Mass Index; EPIC: European Prospective Investigation of Cancer; FFQ: food frequency questionnaires; F&V: fruits and vegetables; MD: Mediterranean Diet; RCTs: randomized controlled trials; USPSTF: U.S. Preventive Services Task Force.

## Competing interests

The authors declared that they have no competing interests.

## Authors’ contributions

JCM, MW, PJM and FFS conceived the study. JL, NH, PJM, TDM, AA, LE LR, FFS, MW and JCM designed the study, while PJM, TDM, AA, LR, FFS, MW and JCM oversaw its implementation. LE performed database searches. JL coordinated and performed study selection, data extraction and quality assessment. JL, MW and JCM planned and interpreted, and JL conducted the meta-analyses and meta-regression. JL and JCM wrote the first draft. All authors reviewed the study findings, and read and approved the final version before submission.

## Pre-publication history

The pre-publication history for this paper can be accessed here:

http://www.biomedcentral.com/1741-7015/12/60/prepub

## Supplementary Material

Additional file 1: Box S1 Search Strategy for systematic review of effectiveness of dietary interventions among adults of retirement age: database searched - Ovid MEDLINE(R) (1950 to April Week 3 2013). **Table S1.** Characteristics of RCTs included in systematic review of effectiveness of dietary interventions among adults of retirement age. **Table S2.** Features of dietary interventions among adults of retirement age included in the systematic review. **Figure S1.** RCTs reporting overall fruit and vegetable intakes by body mass index among people of retirement age. **Figure S2.** RCTs reporting fruit intakes by body mass index among people of retirement age. **Figure S3.** RCTs reporting vegetable intakes by body mass index among people of retirement age. **Figure S4.** RCTs reporting fish intake among people of retirement age. **Figure S5.** RCTs reporting meat intake among people of retirement age. **Figure S6.** Meta-regression analysis of effects of length of follow-up on fruit and vegetable intake. Slope = 0.72, Q = 0.91, d.f. = 1, *P* = 0.34. The circle size reflects the weight that a study obtained in the meta-regression. **Figure S7.** Meta-regression analysis of effects of study sample size on fruit and vegetable intake. Slope = 0.002; Q = 0.13, d.f. = 1, *P* = 0.72. The circle size reflects the weight that a study obtained in the meta-regression. **Figure S8.** Meta-regression analysis of effects of study retention rate on fruit and vegetable intake. Slope = 0.77; Q = 0.39, d.f. = 1, *P* = 0.53. The circle size reflects the weight that a study obtained in the meta-regression. **Figure S9.** Meta-regression analysis of effects of baseline F&V intakes on fruit and vegetable intake. Slope = 0.14; Q = 1.30, d.f. = 1, *P* = 0.25. The circle size reflects the weight that a study obtained in the meta-regression. **Figure S10.** Prisma checklist. Click here for file
