# Supplementary material for: Effectiveness of dietary interventions among adults of retirement age: a systematic review and meta-analysis of randomized controlled trials
Source: BMC Med. 2014 Apr 8;12:60. doi: 10.1186/1741-7015-12-60 (PMC4021978; doi:10.1186/1741-7015-12-60)
Supplement: Additional file 1: Box S1 — Search Strategy for systematic review of effectiveness of dietary interventions among adults of retirement age: database searched - Ovid MEDLINE(R) (1950 to April Week 3 2013). Table S1. Characteristics of RCTs included in systematic review of effectiveness of dietary interventions among adults of retirement age. Table S2. Features of dietary interventions among adults of retirement age included in the systematic review. Figure S1. RCTs reporting overall fruit and vegetable intakes by body mass index among people of retirement age. Figure S2. RCTs reporting fruit intakes by body mass index among people of retirement age. Figure S3. RCTs reporting vegetable intakes by body mass index among people of retirement age. Figure S4. RCTs reporting fish intake among people of retirement age. Figure S5. RCTs reporting meat intake among people of retirement age. Figure S6. Meta-regression analysis of effects of length of follow-up on fruit and vegetable intake. Slope = 0.72, Q = 0.91, d.f. = 1, P = 0.34. The circle size reflects the weight that a study obtained in the meta-regression. Figure S7. Meta-regression analysis of effects of study sample size on fruit and vegetable intake. Slope = 0.002; Q = 0.13, d.f. = 1, P = 0.72. The circle size reflects the weight that a study obtained in the meta-regression. Figure S8. Meta-regression analysis of effects of study retention rate on fruit and vegetable intake. Slope = 0.77; Q = 0.39, d.f. = 1, P = 0.53. The circle size reflects the weight that a study obtained in the meta-regression. Figure S9. Meta-regression analysis of effects of baseline F&V intakes on fruit and vegetable intake. Slope = 0.14; Q = 1.30, d.f. = 1, P = 0.25. The circle size reflects the weight that a study obtained in the meta-regression. Figure S10. Prisma checklist. [file 1741-7015-12-60-S1.docx]

**Supplementary material**

**Box S1**. Search Strategy for systematic review of interventions promoting components of a Mediterranean diet among adults of retirement age: database searched - Ovid MEDLINE(R) (1950 to April Week 3 2013).

| 1 Diet, Mediterranean/ |
| --- |
| 2 cretan diet.mp. |
| 3 mediterranean.mp. |
| 4 exp Diet/ |
| 5 diet.mp. |
| 6 exp Food/ |
| 7 exp Fruit/ |
| 8 Vegetables/ |
| 9 fish.mp. |
| 10 exp Seafood/ |
| 11 red meat.mp. |
| 12 olive oil.mp. |
| 13 red wine.mp. |
| 14 4 or 5 |
| 15 3 or 6 or 7 or 8 or 9 or 10 or 11 or 12 or 13 |
| 16 14 and 15 |
| 17 1 or 2 or 16 |
| 18 randomized controlled trial.pt. |
| 19 controlled clinical trial.pt. |
| 20 randomized.ab. |
| 21 placebo.ab. |
| 22 clinical trials as topic.sh. |
| 23 randomly.ab. |
| 24 trial.ti. |
| 25 18 or 19 or 20 or 21 or 22 or 23 or 24 |
| 26 exp animals/ not humans.sh. |
| 27 25 not 26 |
| 28 17 and 27 |
| 29 older$.ti,ab. |
| 30 elder$.ti,ab. |
| 31 senior$.ti,ab. |
| 32 retir$.ti,ab. |
| 33 Retirement/ |
| 34 aged/ or middle aged/ |
| 35 ag?ing.ti,ab. |
| 36 or/29-35 |
| 37 28 and 36 |

**Table S1.** Characteristics of RCTs included in systematic review of interventions promoting components of a Mediterranean diet among adults of retirement age.

| **Author/ year** | **Sample population** | **Mean Age (years)** | **Mean BMI Gender (% female)** | **Participants health status** | **Baseline Sample size** | **Retention Rate (%)** | **Intervention** | **Control group** |
| --- | --- | --- | --- | --- | --- | --- | --- | --- |
| Tilley et al, 1999[[1](#_ENREF_1)] | USA  Next Step Trial | 57±12 | 26.9±3.9  F/M (3) | High risk CRC | Intervention= 1578  Control= 1899 | 86·3  81·0 | Nutrition intervention designed to decrease fat intake and increase consumption of fibre, fruits, and vegetables.  Five nutrition classes, printed material and personalised dietary feedback | No intervention |
| Bemelmans et al, 2004[[2](#_ENREF_2)] | Netherlands  MARGARIN project | 55±10 | 29.8 ± 3.9  F/M (63) | High CVD risk | Intervention= 103  Control= 163 | 80·5  87·1 | Mediterranean diet  Intensive group education: three meetings of 2 hours each plus four booklets with core information about the education programme | Leaflet Dutch diet guidelines |
| Bowen et al, 2009[[3](#_ENREF_3)] | USA  Eating for life project - religious organizations | 54±16 | ---  F/M (85) | Healthy | Intervention= 1099  Control=1076 | 100  100 | Multilevel package to lower fat and increase fruit and vegetable consumption. | Minimal intervention |
| Little et al, 2004[[4](#_ENREF_4)] | UK | 55±10 | ---  F/M (44) | High BP on a single occasion | Intervention= 145  Control= 151 | 83.4  82·1 | A brief intervention using prompt sheets for high fruit, vegetable, fibre; and low fat to be used when shopping/cooking; and provision of low sodium, high potassium salt. | Leaflet (avoid salty foods) |
| Marcus et al, 2001[[5](#_ENREF_5)] | USA | 54 | ---  F/M (80) | Callers to the NCI cancer information service | Intervention= 801  Control= 905 | 58·9  59·5 | Brief telephone intervention to increase Fruit and  Vegetable Intake | No intervention |
| Takahashi et al, 2006[[6](#_ENREF_6)] | Japan  Rural villages | 56 | 23.6  F/M (67) | Healthy | Intervention= 274  Control= 276 | 97·4  96·7 | Dietary advice to increase the intake of vitamin C and Carotene, and of fruits and vegetables.  Moderate intensity tailored dietary counselling | No intervention |
| Estruch et al, 2013 [[7](#_ENREF_7)] | Spain | 67±6 | 29.9 ±3.8  F/M (53) | >3 CVD factors | Intervention= 4997  Control= 2450 | 95  89 | Mediterranean diet  Groups sessions + access to dietitian | Leaflet low-fat diet |
| Campbell et al, 1999[[8](#_ENREF_8)] | USA  Rural African-American | 54 | --  F/M (73) | Low risk | Intervention= 1198  Control= 1321 | 77·3 | Increasing fruit and vegetable intake.  Multicomponent: Tailored bulletins, educational sessions, F&V availability at church, etc | Delayed intervention |
| Stevens et al, 2003[[9](#_ENREF_9)] | USA | 54±7 | 30.2±7.1  F (100) | Low risk | Intervention= 308  Control= 308 | 89  85 | Decrease consumption of fat and increase consumption of fruits and vegetables  Computer assisted intervention | No intervention |
| Howard et al, 2006[[10](#_ENREF_10)] | USA  WHIDMT intervention  Postmenopausal Women | 62±7 | 29.7  F (100) | Healthy | Intervention= 19541  Control= 29 294 | 90·4  91·1 | A dietary pattern lower in fat and higher in vegetables, fruit, and grains  Eighteen group-sessions/yr | Usual care (minimum interference) |
| Wolf et al, 2009[[11](#_ENREF_11)] | USA  Immigrant Black men | 55±6 | --  M (100) | Low risk | Intervention= 240  Control= 239 | 87·8  88·1 | Promote awareness and adoption of fruit and vegetable recommendations.  Tailored telephone education | No intervention |
| Walker et al, 2009[[12](#_ENREF_12)] | USA  Rural women | 58±5 | 30.7 ±6.8  F (100) | Low risk | Intervention= 115  Control= 110 | 92·2  99 | Intervention on physical activity and healthy eating (fruits, vegetables, and whole grain products).  Tailored newsletter | Generic newsletter |
| Merrill et al, 2009[[13](#_ENREF_13)] | USA  CHIP intervention  Women | 57±9 | 33.9  F/M (35) | Healthy | Intervention= 69  Control= 50 | 91·3  96 | Adopting a more plant food-based diet that emphasizes whole grains, legumes, vegetables, and fresh fruits. In addition, the diet was low in fat, animal protein, sugar, and salt, very low in cholesterol, and high in fibre.  Intensive 40 hr course plus a health promotion textbook and workbooks, and access to scheduled shopping tours and cooking demonstrations. | No intervention |
| Lapointe et al, 2010[[14](#_ENREF_14)] | Canada  Postmenopausal women | 57±5 | 30  F (100) | Postmenopausal women | Intervention= 35  Control= 33 | 91·4  93·9 | Dietary intervention promoting high intakes of fruits and vegetables.  3 group-sessions 10 individual-sessions | Limit high fat food3 group-sessions 10 individual-sessions |
| Lanza et al, 2001[[15](#_ENREF_15)] | USA  PPT  Men and women | 62±10 | 27.6 ± 3.2  F/M (35) | Participants diagnosed with polyps in the previous 6 months | Intervention= 923  Control= 916 | 89  88 | Increase fruits and vegetables and reduce fat.  Intensive nutrition education and counselling program | No intervention |
| Werkman et al. 2010[[16](#_ENREF_16)] | Netherlands  Recent retirees | 60±3 | 26.7±3.6  M  (study included few women, but published results include only men) | Healthy | Intervention=209  Control =204 | 91 | Lifestyle intervention on physical activity and diet.  Increase dietary fibre by increasing consumption of whole grain foods, fruits and vegetables; and reduce fat intake.  Content: Printed and computer tailored modules to improve PA and diet. | Newsletters and general information about the study |
| Van keulen et al. 2011[[17](#_ENREF_17)] | Netherlands | 57±7 | 27.4±4.6  F/M (45) | 52% hypertensive subjects | Multiple arms = 1205  Control 404 | 73 | Reduce fat intake, increase fruit and vegetable intake  Tailored print communication; or Telephone motivational interviewing; or Combination of these | No intervention |
| Kanaya et al. 2012[[18](#_ENREF_18)] | USA  Ethnic minorities and low SES adults | 56±16 | 30.1 ± 5.3  F/M (73) | Subjects at risk of DM | Intervention =113  Control =117 | 100 | Education and skills training to modify diet and physical activity through primarily telephone-based counselling (12 calls) with 2 in-person sessions and 5 optional group workshops. | Waiting list intervention |
| Wright et al. 2011[[19](#_ENREF_19)] | Australia | 56±7 | 30  F/M (52) | Participants having one or more CVD risk factors | Intervention =116  Control =62 | 100 | Reduce saturated fat related intake and behaviours and increase intake of fruit vegetables and cereal and grain food (in particular whole grains).  1) tailored, iterative, printed dietary feedback with 3 instalments mail-delivered over a 3-month period  2) small group nutrition education sessions consisting of 2 90-minute dietitian-led sessions. | Waiting list intervention |
| Wilcox et al. 2013[[20](#_ENREF_20)] | USA  African Methodist Episcopal churches | 54±14 | 32.9±7.8  F/M (24.3) | Subjects at risk of CVD | Intervention =749  Control = 508 | 62 | Increase physical activity and fruit and vegetable consumption.  15-month intervention targeting organizational and environmental changes consistent with the structural ecologic model. A community-based participatory research approach guided intervention development. Intervention churches attended a full-day committee training and a full-day cook training. They also received a stipend and 15 months of mailings and technical assistance calls to support intervention implementation. | Delayed intervention |
| Panunzio et al. 2011[[21](#_ENREF_21)] | Italy | 55±1 | --  F/M (43) | Healthy | Intervention =40  Control = 40 | 76·3 | Mediterranean diet.  Intensive phase of 15 weeks involving 10 modules of a training course on lifestyle-related themes.  Consolidation phase of 10 weeks consisting of 4 meetings directed to reinforce the changes achieved. | No intervention |
| Coates et al. 1999[[22](#_ENREF_22)] | USA | 60 | --  F (100) | Healthy | Intervention =1324  Control = 883 | -- | Reduce fat intake, increase fruit and vegetable intake  Delivered intervention in group sessions that met weekly for 6 weeks, biweekly for 6 weeks, monthly for 9 months, and then quarterly. | Dietary allowances for Americans booklet |

**Table S2.** Features of dietary interventions promoting components of a Mediterranean diet among adults of retirement age included in the systematic review

| **Study** | **Provider** | **Format** | **Setting** | **Intensity** | **Duration** |
| --- | --- | --- | --- | --- | --- |
| Marcus et al. 2001[[5](#_ENREF_5)] | Information specialists at CIS centre | Telephone | Telephone interviews  (when the patient called the Cancer Information Service) | Baseline interview, then follow up at 4 weeks, 4 months & 12 months. Each phone call between 5-7 mins long | 12 months  Telephone interview at baseline, 4 weeks, 4 months & 12 months.  Participants recruited over 12 days between 4th-19th June 1996 |
| Little et al. 2004[[4](#_ENREF_4)] | Nurse | Face to face | GP practices | Three face to face sessions; at baseline, 4 weeks and 6 months | 6 months |
| Lapointe et al. 2010[[14](#_ENREF_14)] | Dietitian | Face to face – group and individual sessions | Unclear | 3 group sessions and ten individual sessions with a registered dietitian in 6 months | 6 months |
| Lanza et al. 2001[[15](#_ENREF_15)] | Nutritionist | Face to face – individual & group  vs  general dietary guidelines from the National Dairy Council | Clinical centres | They had more than 50 hours of individual and group counselling sessions over 4 yrs | 4 years |
| Campbell et al. 1999[[8](#_ENREF_8)] | Trained interviewers  Mailed intervention  Pastors (sermons) | Posted printed material plus reinforcing messages from pastors | Home and Church | Monthly printed material; sermons at church reinforcing messages | 20 months |
| Howard et al 2006[[10](#_ENREF_10)] | Trained Nutritionists | Face to face group sessions | Clinical centres | 18 group sessions in year 1. Quarterly maintenance sessions thereafter (5 yrs) | 1 year |
| Stevens et al. 2003[[9](#_ENREF_9)] | Health counsellors | 20 min interactive touch-screen computer based | Clinical centre | Two 45-min counselling sessions (One of these including a 20-min interactive touch-screen computer based)  Two brief –telephone calls | 6-9 weeks |
| Takahashi et al 2006[[6](#_ENREF_6)] | Dietitian | Face to face counselling  Posted newsletters  1 x group lecture | Unclear | 2 x dietary counselling sessions (5 months o apart)  2 x newsletters  1 x group lecture (halfway) | 10 months |
| Tilley et al 1999[[1](#_ENREF_1)] | Unclear | Face to face groups classes | Worksite | (Year 1)  5 nutrition classes  Mailed self-help material  (Year 2)  Worksite posters, personalised feedback on FFQ | 2 years |
| Walker et al 2009[[12](#_ENREF_12)] | Nurse involved in supervised self-report questionnaires | Mail | Community/home | 18 newsletters in 12 months (every 2 weeks during first 6 months; every 4 weeks months 7-12)  Feedback on diet? PA at 1, 3, 6 and 9 months  Motivational devices: Food pyramid magnets for fridges and pedometers | 12 months |
| Wolf et al. 2009[[11](#_ENREF_11)] | Health educators | Two telephone calls  Mailed brochure | Community/Home | Two telephone calls within a month. One educational call; one follow-up call. | 1 months |
| Bowen et al. 2009[[3](#_ENREF_3)] | Healthy eating coordinators | Mailed material  Social activities  Healthy eating sessions | Church/ community | Control group received standard educational materials.  Intervention group received culturally targeted self-help nutrition and PA materials, and 4 telephone counselling calls based on motivational interviewing delivered over the course of I year | 1 year |
| Merril et al. 1999[[13](#_ENREF_13)] | Dietitians, medical professionals e.g. nurses, Coronary Health Improvement Project (CHIP) staff | Face to face  scheduled shopping tours & cooking demos  Textbooks and workbooks | Clinical centre | Participants met for 4 weeks—four times each week for 2 hours on the CHIP study. Dietitians & medical professionals spoke weekly. Participants had access to scheduled shopping tours & cooking demos by the dietitian | 1 months |
| Estruch et al. 2013[[7](#_ENREF_7)] | Dietitian | Individual interviews  group sessions with written shopping lists meal plans etc | Clinical centres | Quarterly interviews  Quarterly group sessions  1 x follow up medical assessment | 12 months  Quarterly interviews and group sessions  1 x follow-up medical assessment |
| Bemelmans et al. 2004[[2](#_ENREF_2)] | Dietitian for intensive group approach vs GP for usual care | Face to face –  group sessions by Dietitian vs Usual care in the form of a leaflet | Unclear | 3 group sessions of 2 hours each | Baseline march 2008  Follow-up at 16 and 52 weeks.  Plans for 105, 146 and 208 week follow-up |
| Werkman et al. 2010[[16](#_ENREF_16)] | Unclear | Internet  And printed material | unclear | Five programme modules were provided to participants  of the intervention group during the 1-yr intervention. Participants could freely choose to make use of the modules or not. In addition, the intervention group received newsletters every 2-3 months that contained study information, information about diet and physical activity and encouragements to use the modules. | 12 months |
| Van keulen et al. 2011[[17](#_ENREF_17)] | Trained interviewer counsellors | Tailored print communication;  Telephone motivational interviewing;  Combination of these | Mail and/or telephone delivered intervention | Tailored print communication: 4 tailored letters (#1 and #3 focused on PA and #2 and #4 on fruit and vegetable consumption).  Telephone motivational interviewing: 4 telephone calls based on motivational interviewing. Participants chose the order of the conversation topics in #1 and #3; if PA was preferred in #1, fruit and vegetable consumption was discussed in #2, and vice versa.  Combination of these: 2 tailored print letters and 2 telephone motivational interviews; #1 (letter) and #2 (interview) addressed PA, #3 (letter) and #4 (interview) focused on fruit and vegetable consumption. | 73 weeks |
| Kanaya et al. 2012[[18](#_ENREF_18)] | Trained health department counsellors | Telephone-based counselling, 2 in-person sessions and 5 optional group workshops. | Community | The program consisted of 19 possible “contacts” for a total of 15 possible hours: 1 introductory session, which included a program binder; 1 in-person planning session; 12 telephone counselling calls (10 in active phase, 2 in maintenance phase); and 5 group workshops. | 12 months |
| Wright et al. 2011[[19](#_ENREF_19)] | Dietitian | 1) tailored, iterative, printed dietary feedback  2) small group nutrition education sessions dietitian-led | University seminar rooms | 1) tailored, iterative, printed dietary feedback (TF) with three instalments mail-delivered over a 3-month period that were re-tailored to most recent assessment of dietary intake, intention to change and assessment of self-adequacy of dietary intake.  2) small group nutrition education sessions (GE): consisting of two 90-minute dietitian-led small group sessions l | 3 months |
| Wilcox et al. 2013[[20](#_ENREF_20)] | Trained members of the community |  | Church | 15-month intervention targeting organizational and environmental changes consistent with the structural ecologic model. Intervention churches attended a full-day committee training and a full-day cook training. They also received a stipend and 15 months of mailings and technical assistance calls to support intervention implementation. | 15 months |
| Panunzio et al. 2011[[21](#_ENREF_21)] | Nutritionist  Psychologists | Face to face – group and individual sessions | Unclear | 2-phases: Intensive phase of 15 weeks and Consolidation phase of 10 weeks.  Intensive phase: 5 weeks involving 10 (1-hr each) modules of a training course on lifestyle-related themes. 5 weeks of experiential “learn by doing” phase. And 5 weeks of 2 (1 hour each) modules per week.  Consolidation phase of 10 weeks consisting of 4 meetings (1 approximately every 17 days) directed to reinforce the changes achieved. | 25 weeks |
| Coates et al. 1999[[22](#_ENREF_22)] | Nutritionist | Face to face – group sessions | Clinic | The intervention was based on the Vanguard Women's Health Trial program modified to include the additional goals. Nutritionists assigned personal fat gram goals to each participant and delivered the intervention in group sessions that met weekly for 6 weeks, biweekly for 6 weeks, monthly for 9 months, and then quarterly. | 18 months |

**Figure S1.** Forest plot of effects of dietary intervention studies by body mass index reporting overall fruit and vegetable intakes among people of retirement age


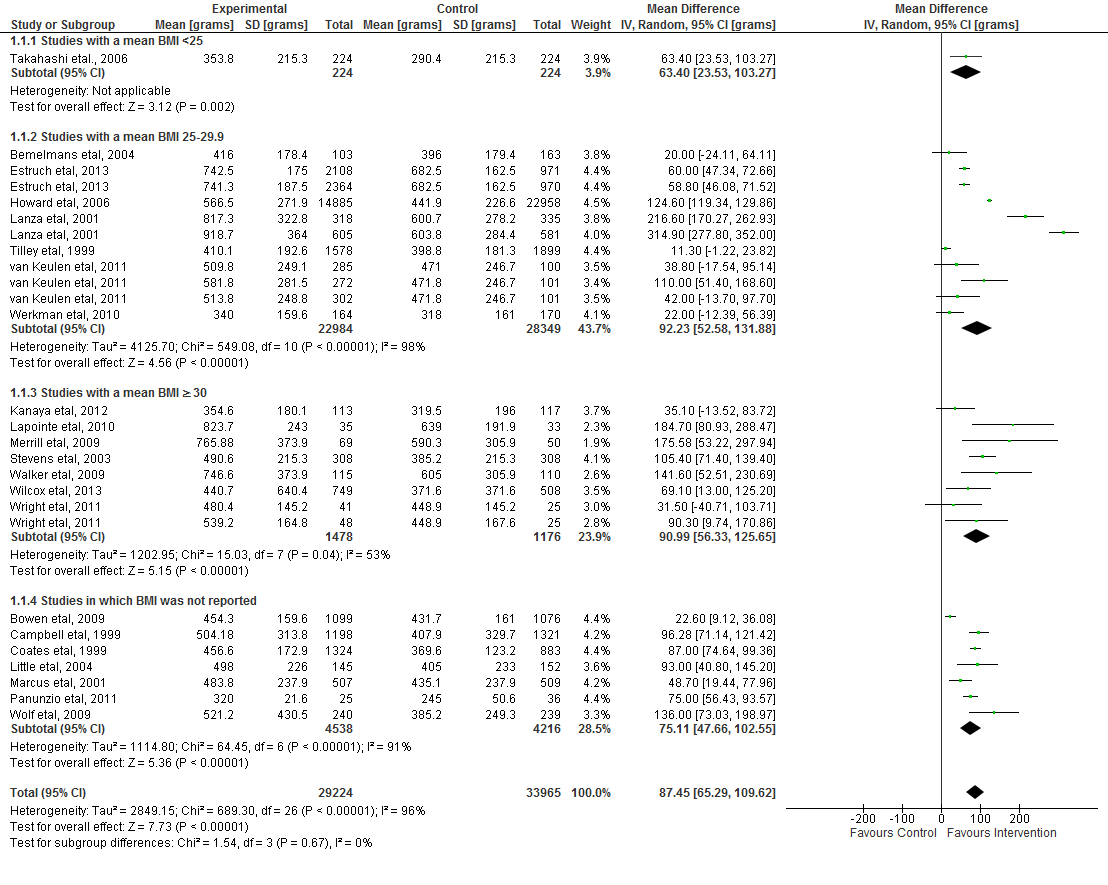


**Figure S2**. Forest plot of effects on fruit intake of dietary intervention studies by body mass index among people of retirement age


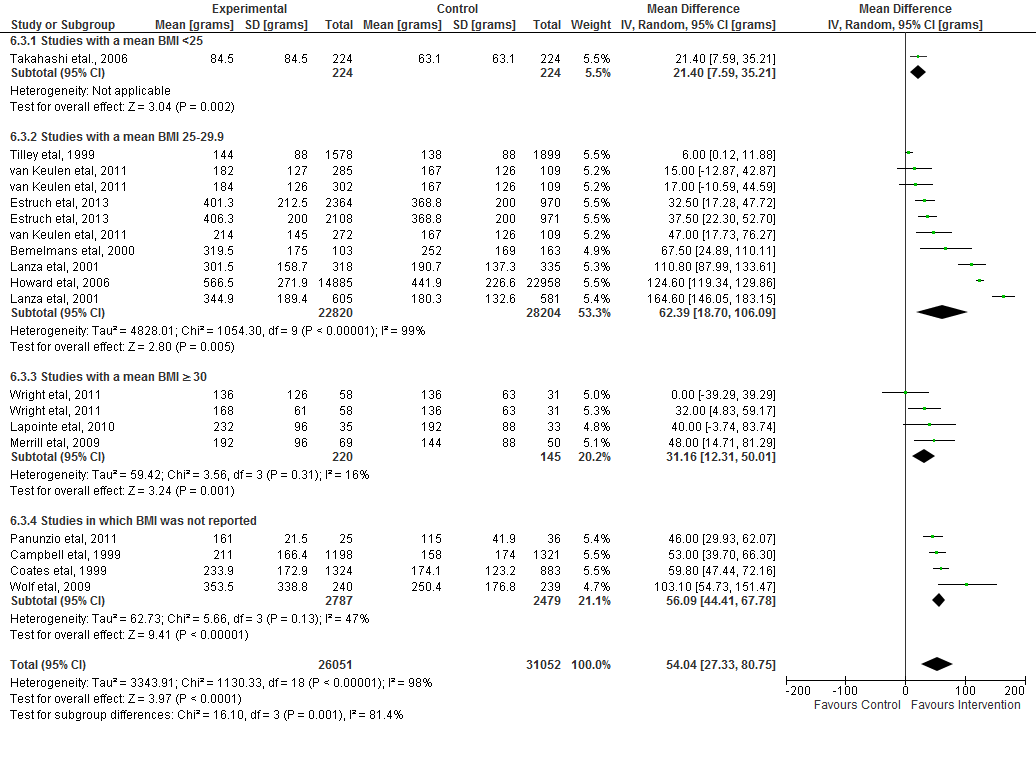


**Figure S3**. Forest plot of effects on vegetable intake of dietary intervention studies by body mass index among people of retirement age


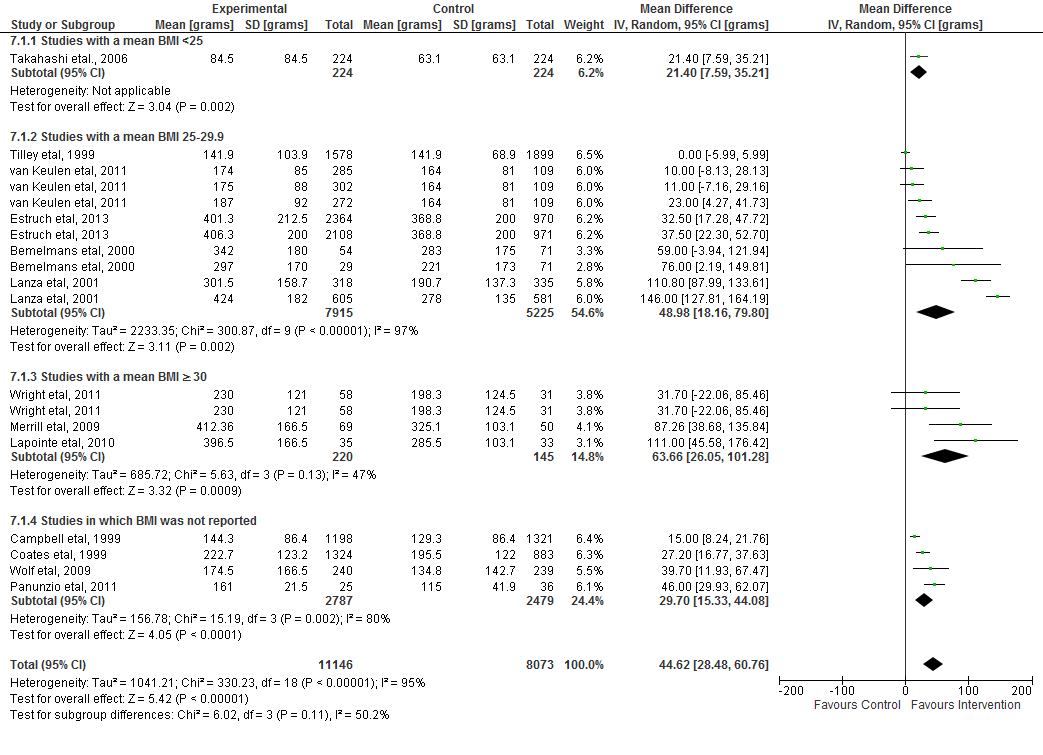


**Figure S4.** Forest plot of effects on fish intake of dietary intervention studies among people of retirement age

**
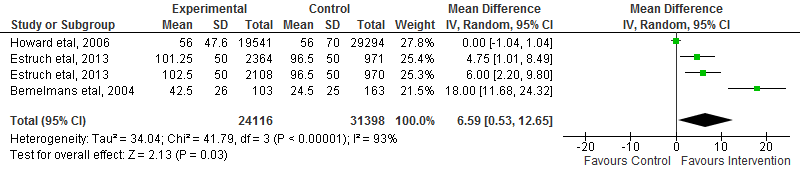
**

**Figure S5.** Forest plot of effects on meat intake of dietary intervention studies among people of retirement age

**
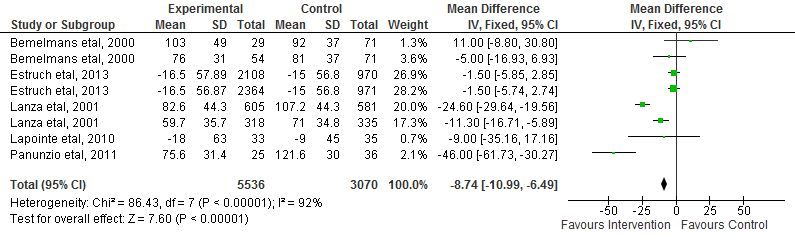
**

**Figure S6**. Relationship between length of follow-up and change in fruit and vegetable consumption in response to intervention. Slope = 0·72, Q = 0·91, d.f. = 1, p = 0·34. The circle size reflects the weight that a study obtained in the meta-regression.


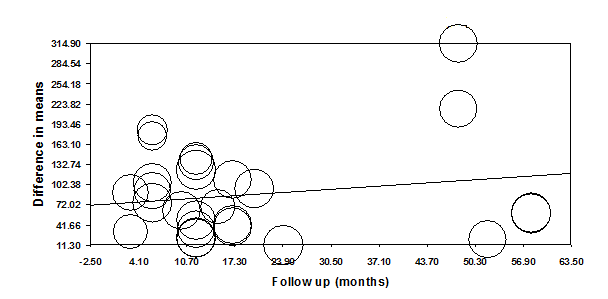


**Figure S7**. Relationship between study sample size and change in fruit and vegetable consumption in response to intervention. Slope = 0·002; Q = 0·13, d.f. = 1, p = 0·72. The circle size reflects the weight that a study obtained in the meta-regression.


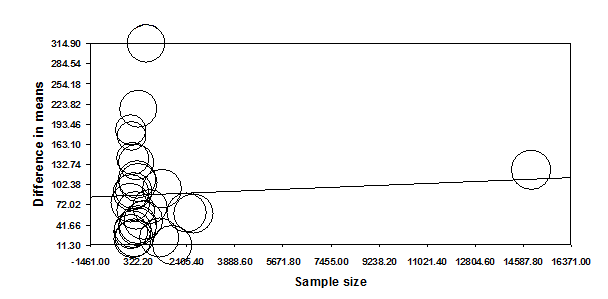


**Figure S8**. Relationship between study attrition and change in fruit and vegetable consumption in response to intervention. Slope = 0·77; Q = 0·39, d.f. = 1, p = 0·53. The circle size reflects the weight that a study obtained in the meta-regression.


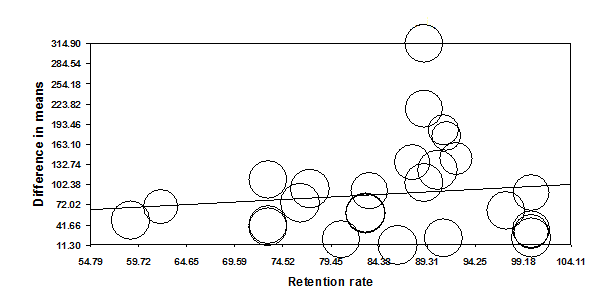


**Figure S9.** Relationship between baseline F&V intake (g/day) and change in fruit and vegetable consumption in response to intervention. Slope = 0·14; Q = 1·30, d.f. = 1, p = 0·25. The circle size reflects the weight that a study obtained in the meta-regression.

**
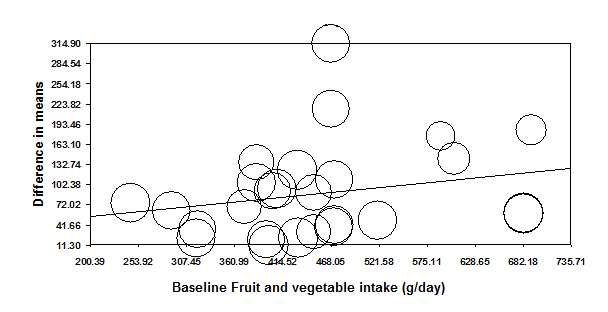
**

**Figure S10**. Prisma checklist.

| **Section/topic** | **#** | **Checklist item** | **Reported on page #** |
| --- | --- | --- | --- |
| **TITLE** | | |  |
| Title | 1 | Identify the report as a systematic review, meta-analysis, or both. | 1 |
| **ABSTRACT** | | |  |
| Structured summary | 2 | Provide a structured summary including, as applicable: background; objectives; data sources; study eligibility criteria, participants, and interventions; study appraisal and synthesis methods; results; limitations; conclusions and implications of key findings; systematic review registration number. | 2-3 |
| **INTRODUCTION** | | |  |
| Rationale | 3 | Describe the rationale for the review in the context of what is already known. | 4-5 |
| Objectives | 4 | Provide an explicit statement of questions being addressed with reference to participants, interventions, comparisons, outcomes, and study design (PICOS). | 5 |
| **METHODS** | | |  |
| Protocol and registration | 5 | Indicate if a review protocol exists, if and where it can be accessed (e.g., Web address), and, if available, provide registration information including registration number. | 6 |
| Eligibility criteria | 6 | Specify study characteristics (e.g., PICOS, length of follow-up) and report characteristics (e.g., years considered, language, publication status) used as criteria for eligibility, giving rationale. | 6-7 |
| Information sources | 7 | Describe all information sources (e.g., databases with dates of coverage, contact with study authors to identify additional studies) in the search and date last searched. | 6 |
| Search | 8 | Present full electronic search strategy for at least one database, including any limits used, such that it could be repeated. | suppl material |
| Study selection | 9 | State the process for selecting studies (i.e., screening, eligibility, included in systematic review, and, if applicable, included in the meta-analysis). | 8 |
| Data collection process | 10 | Describe method of data extraction from reports (e.g., piloted forms, independently, in duplicate) and any processes for obtaining and confirming data from investigators. | 8 |
| Data items | 11 | List and define all variables for which data were sought (e.g., PICOS, funding sources) and any assumptions and simplifications made. | 8 |
| Risk of bias in individual studies | 12 | Describe methods used for assessing risk of bias of individual studies (including specification of whether this was done at the study or outcome level), and how this information is to be used in any data synthesis. | 8 |
| Summary measures | 13 | State the principal summary measures (e.g., risk ratio, difference in means). | 8 |
| Synthesis of results | 14 | Describe the methods of handling data and combining results of studies, if done, including measures of consistency (e.g., I^2^) for each meta-analysis. | 9 |

| **Section/topic** | **#** | **Checklist item** | **Reported on page #** |
| --- | --- | --- | --- |
| Risk of bias across studies | 15 | Specify any assessment of risk of bias that may affect the cumulative evidence (e.g., publication bias, selective reporting within studies). | 9 |
| Additional analyses | 16 | Describe methods of additional analyses (e.g., sensitivity or subgroup analyses, meta-regression), if done, indicating which were pre-specified. | 9 |
| **RESULTS** | | |  |
| Study selection | 17 | Give numbers of studies screened, assessed for eligibility, and included in the review, with reasons for exclusions at each stage, ideally with a flow diagram. | 24 |
| Study characteristics | 18 | For each study, present characteristics for which data were extracted (e.g., study size, PICOS, follow-up period) and provide the citations. | suppl material |
| Risk of bias within studies | 19 | Present data on risk of bias of each study and, if available, any outcome level assessment (see item 12). |  |
| Results of individual studies | 20 | For all outcomes considered (benefits or harms), present, for each study: (a) simple summary data for each intervention group (b) effect estimates and confidence intervals, ideally with a forest plot. | 26-28 |
| Synthesis of results | 21 | Present results of each meta-analysis done, including confidence intervals and measures of consistency. |  |
| Risk of bias across studies | 22 | Present results of any assessment of risk of bias across studies (see Item 15). |  |
| Additional analysis | 23 | Give results of additional analyses, if done (e.g., sensitivity or subgroup analyses, meta-regression [see Item 16]). | 29 |
| **DISCUSSION** | | |  |
| Summary of evidence | 24 | Summarize the main findings including the strength of evidence for each main outcome; consider their relevance to key groups (e.g., healthcare providers, users, and policy makers). | 13 |
| Limitations | 25 | Discuss limitations at study and outcome level (e.g., risk of bias), and at review-level (e.g., incomplete retrieval of identified research, reporting bias). | 14-15 |
| Conclusions | 26 | Provide a general interpretation of the results in the context of other evidence, and implications for future research. | 16-17 |
| **FUNDING** | | |  |
| Funding | 27 | Describe sources of funding for the systematic review and other support (e.g., supply of data); role of funders for the systematic review. | 18 |

**Table S3**. Evidence from systematic reviews and meta-analyses on the health effects of increased consumption of fruits and vegetables

| **Reference** | **No of studies included** | **Type of studies** | **Food group evaluated** | **Change evaluated** | **Outcome** | **RR (95%CI)** |
| --- | --- | --- | --- | --- | --- | --- |
| **Dauchet et al. 2005[**[**23**](#_ENREF_23)**]** | 7 | Cohorts | Fruit | Per 106g increase | Stroke | 0·89 (0·85 to 0·93) |
|  |  |  | Fruit and vegetable |  |  | 0·95 (0·92 to 0·97) |
|  |  |  | Vegetables |  |  | 0·97 (0·92 to 1·02) |
| **Dauchet et al. 2006[**[**24**](#_ENREF_24)**]** | 9 | Cohorts | Fruit and vegetable | Per 106g increase | CHD | 0·96 (0·93 to 0·99) |
|  |  |  | Fruit |  |  | 0·93 (0·89 to 0·96) |
| **He et al., 2006[**[**25**](#_ENREF_25)**]** | 8 | Cohorts | Fruit and vegetable | <3 vs 3-5 servings | Stroke | 0·89 (0·83 to 0·97) |
| **He et al., 2007[**[**26**](#_ENREF_26)**]** | 13 | Cohorts | Fruit and vegetable | <3 vs 3-5 servings | CHD | 0·93 (0·86 to 1·00) |
| **Pavia et al., 2006[**[**27**](#_ENREF_27)**]** | 16 | 15 Case-control  1 Cohort | Fruit | Per 1 portion increase | Oral cancer | 0·51 (0·40 to 0·65) |
|  |  |  | Vegetables |  |  | 0·50 (0·38 to 0·65) |
| **Lunet et al., 2005[**[**28**](#_ENREF_28)**]** | 13 | Cohorts | Fruit | Per 100g increase | Gastric cancer | 0·89 (0·78 to 1·02) |
|  |  |  | Vegetables | Per 100g increase |  | 0·98 (0·86 to 1·13) |
| **Reference** | **No of studies included** | **Type of studies** | **Food group evaluated** | **Change evaluated** | **Outcome** | **HR (95%CI)** |
| **Carter et al., 2010[**[**29**](#_ENREF_29)**]** | 6 | Cohort | Fruit and vegetable | Per 1·35 servings increase | Diabetes | 0·86 (0·77 to 0·97) |
| **Cooper et al., 2012[**[**30**](#_ENREF_30)**]** | 8 | Cohort | Fruit and vegetable |  | Diabetes | 0.90 (0.80 to 1.01) |
|  |  |  | Fruit |  |  | 0.89 (0.76 to 1.04) |
|  |  |  | Vegetables |  |  | 0.94 (0.84 to 1.05) |

**References**

1. Tilley BC, Glanz K, Kristal AR, Hirst K, Li S, Vernon SW, Myers R: **Nutrition intervention for high-risk auto workers: results of the Next Step Trial**. *Preventive medicine* 1999, **28**(3):284-292.

2. Bemelmans WJE, Broer J, Hulshof KFAM, Siero FW, May JF, Meyboom-de Jong B: **Long-term effects of nutritional group education for persons at high cardiovascular risk**. *The European Journal of Public Health* 2004, **14**(3):240-245.

3. Bowen DJ, Beresford SA, Christensen CL, Kuniyuki AA, McLerran D, Feng Z, Hart A, Jr., Tinker L, Campbell M, Satia J: **Effects of a multilevel dietary intervention in religious organizations**. *American journal of health promotion : AJHP* 2009, **24**(1):15-22.

4. Little P, Kelly J, Barnett J, Dorward M, Margetts B, Warm D: **Randomised controlled factorial trial of dietary advice for patients with a single high blood pressure reading in primary care**. *BMJ* 2004, **328**(7447):1054.

5. Marcus AC, Heimendinger J, Wolfe P, Fairclough D, Rimer BK, Morra M, Warnecke R, Himes JH, Darrow SL, Davis SW *et al*: **A randomized trial of a brief intervention to increase fruit and vegetable intake: a replication study among callers to the CIS**. *Preventive medicine* 2001, **33**(3):204-216.

6. Takahashi Y, Sasaki S, Okubo S, Hayashi M, Tsugane S: **Blood pressure change in a free-living population-based dietary modification study in Japan**. *Journal of hypertension* 2006, **24**(3):451-458.

7. Estruch R, Ros E, Salas-Salvado J, Covas MI, Corella D, Aros F, Gomez-Gracia E, Ruiz-Gutierrez V, Fiol M, Lapetra J *et al*: **Primary prevention of cardiovascular disease with a Mediterranean diet**. *The New England journal of medicine* 2013, **368**(14):1279-1290.

8. Campbell MK, Demark-Wahnefried W, Symons M, Kalsbeek WD, Dodds J, Cowan A, Jackson B, Motsinger B, Hoben K, Lashley J *et al*: **Fruit and vegetable consumption and prevention of cancer: the Black Churches United for Better Health project**. *American journal of public health* 1999, **89**(9):1390-1396.

9. Stevens VJ, Glasgow RE, Toobert DJ, Karanja N, Smith KS: **One-year results from a brief, computer-assisted intervention to decrease consumption of fat and increase consumption of fruits and vegetables**. *Preventive medicine* 2003, **36**(5):594-600.

10. Howard BV, Manson JE, Stefanick ML, Beresford SA, Frank G, Jones B, Rodabough RJ, Snetselaar L, Thomson C, Tinker L *et al*: **Low-fat dietary pattern and weight change over 7 years: the Women's Health Initiative Dietary Modification Trial**. *JAMA : the journal of the American Medical Association* 2006, **295**(1):39-49.

11. Wolf RL, Lepore SJ, Vandergrift JL, Basch CE, Yaroch AL: **Tailored telephone education to promote awareness and adoption of fruit and vegetable recommendations among urban and mostly immigrant black men: a randomized controlled trial**. *Preventive medicine* 2009, **48**(1):32-38.

12. Walker SN, Pullen CH, Boeckner L, Hageman PA, Hertzog M, Oberdorfer MK, Rutledge MJ: **Clinical trial of tailored activity and eating newsletters with older rural women**. *Nursing research* 2009, **58**(2):74-85.

13. Merrill RM, Aldana SG: **Consequences of a plant-based diet with low dairy consumption on intake of bone-relevant nutrients**. *J Womens Health (Larchmt)* 2009, **18**(5):691-698.

14. Lapointe A, Weisnagel SJ, Provencher V, Begin C, Dufour-Bouchard AA, Trudeau C, Lemieux S: **Using restrictive messages to limit high-fat foods or nonrestrictive messages to increase fruit and vegetable intake: what works better for postmenopausal women?** *European journal of clinical nutrition* 2010, **64**(2):194-202.

15. Lanza E, Schatzkin A, Daston C, Corle D, Freedman L, Ballard-Barbash R, Caan B, Lance P, Marshall J, Iber F *et al*: **Implementation of a 4-y, high-fiber, high-fruit-and-vegetable, low-fat dietary intervention: results of dietary changes in the Polyp Prevention Trial**. *The American journal of clinical nutrition* 2001, **74**(3):387-401.

16. Werkman A, Hulshof PJ, Stafleu A, Kremers SP, Kok FJ, Schouten EG, Schuit AJ: **Effect of an individually tailored one-year energy balance programme on body weight, body composition and lifestyle in recent retirees: a cluster randomised controlled trial**. *BMC public health* 2010, **10**:110.

17. van Keulen H, Mesters I, Ausems M, Breukelen G, Campbell M, Resnicow K, Brug J, Vries H: **Tailored Print Communication and Telephone Motivational Interviewing Are Equally Successful in Improving Multiple Lifestyle Behaviors in a Randomized Controlled Trial**. *ann behav med* 2011, **41**(1):104-118.

18. Kanaya AM, Santoyo-Olsson J, Gregorich S, Grossman M, Moore T, Stewart AL: **The Live Well, Be Well Study: A Community-Based, Translational Lifestyle Program to Lower Diabetes Risk Factors in Ethnic Minority and Lower–Socioeconomic Status Adults**. *American journal of public health* 2012, **102**(8):1551-1558.

19. Wright J, Sherriff J, Dhaliwal S, Mamo J: **Tailored, iterative, printed dietary feedback is as effective as group education in improving dietary behaviours: results from a randomised control trial in middle-aged adults with cardiovascular risk factors**. *International Journal of Behavioral Nutrition and Physical Activity* 2011, **8**(1):43.

20. Wilcox S, Parrott A, Baruth M, Laken M, Condrasky M, Saunders R, Dowda M, Evans R, Addy C, Warren TY *et al*: **The faith, activity, and nutrition program: a randomized controlled trial in african-american churches**. *American journal of preventive medicine* 2013, **44**(2):122-131.

21. Panunzio MF, Caporizzi R, Antoniciello A, Cela EP, Ferguson LR, D'Ambrosio P: **Randomized, controlled nutrition education trial promotes a Mediterranean diet and improves anthropometric, dietary, and metabolic parameters in adults**. *Ann Ig* 2011, **23**(1):13-25.

22. Coates RJ, Bowen DJ, Kristal AR, Feng Z, Oberman A, Hall WD, George V, Lewis CE, Kestin M, Davis M *et al*: **The Women's Health Trial Feasibility Study in Minority Populations: Changes in Dietary Intakes**. *American journal of epidemiology* 1999, **149**(12):1104-1112.

23. Dauchet L, Amouyel P, Dallongeville J: **Fruit and vegetable consumption and risk of stroke: a meta-analysis of cohort studies**. *Neurology* 2005, **65**(8):1193-1197.

24. Dauchet L, Amouyel P, Hercberg S, Dallongeville J: **Fruit and vegetable consumption and risk of coronary heart disease: a meta-analysis of cohort studies**. *The Journal of nutrition* 2006, **136**(10):2588-2593.

25. He FJ, Nowson CA, MacGregor GA: **Fruit and vegetable consumption and stroke: meta-analysis of cohort studies**. *Lancet* 2006, **367**(9507):320-326.

26. He FJ, Nowson CA, Lucas M, MacGregor GA: **Increased consumption of fruit and vegetables is related to a reduced risk of coronary heart disease: meta-analysis of cohort studies**. *J Hum Hypertens* 2007, **21**(9):717-728.

27. Pavia M, Pileggi C, Nobile CG, Angelillo IF: **Association between fruit and vegetable consumption and oral cancer: a meta-analysis of observational studies**. *The American journal of clinical nutrition* 2006, **83**(5):1126-1134.

28. Lunet N, Lacerda-Vieira A, Barros H: **Fruit and vegetables consumption and gastric cancer: a systematic review and meta-analysis of cohort studies**. *Nutrition and cancer* 2005, **53**(1):1-10.

29. Carter P, Gray LJ, Troughton J, Khunti K, Davies MJ: **Fruit and vegetable intake and incidence of type 2 diabetes mellitus: systematic review and meta-analysis**. *BMJ* 2010, **341**:c4229.

30. Cooper AJ, Forouhi NG, Ye Z, Buijsse B, Arriola L, Balkau B, Barricarte A, Beulens JWJ, Boeing H, Buchner FL *et al*: **Fruit and vegetable intake and type 2 diabetes: EPIC-InterAct prospective study and meta-analysis**. *European journal of clinical nutrition* 2012, **66**(10):1082-1092.
